# Comparative Study on the Resistance of Beta-Cypermethrin Nanoemulsion and Conventional Emulsion in *Blattella germanica*

**DOI:** 10.3390/toxics11100834

**Published:** 2023-10-02

**Authors:** Yan Shen, Qiong Li, Fujin Fang, Chuanli Yang, Yu Dong, Xiaoqin Li, Zhizhi Luo, Xiaobing Shen

**Affiliations:** 1 Key Laboratory of Environmental Medicine Engineering, Ministry of Education, School of Public Health, Southeast University, Nanjing 210009, China; 230198327@seu.edu.cn (Y.S.); joan_tiny@163.com (Q.L.); 230218461@seu.edu.cn (F.F.); 230229015@seu.edu.cn (C.Y.); 15071232302@163.com (Y.D.); 2 School of Public Health, Southeast University, Nanjing 210009, China; 3 Yangzhou Center for Disease Control and Prevention, Yangzhou 225007, China; lixiaoq1983@163.com (X.L.); shy0514@163.com (Z.L.)

**Keywords:** high-efficiency cypermethrin, nanoemulsion, cetylcholinesterase, glutathione S-transferase, P450-O demethylase

## Abstract

Objective: This study aimed to compare the resistance rates of *Blattella germanica* to beta-cypermethrin nanoemulsion and conventional emulsion and establish reference values via biochemical detection for conventional emulsion. Methods: We conducted experiments using subcultured *Blattella germanica* and applied the micro-drop method for treatment. Subsequently, the activity of metabolic enzymes was measured using spectrophotometry. Profile analysis was employed to study the resistance rates of beta-cypermethrin nanoemulsion and beta-cypermethrin emulsion. Results: The regression equation for the relationship between generation and resistance factor in *Blattella germanica* treated with beta-cypermethrin nanoemulsion was as follows: *y*_1_ = 0.091*x*_1_ + 0.991, with an *r*-value of 0.990 (*F* = 95.184, *p* = 0.01 < 0.05). Similarly, the regression equation for *Blattella germanica* treated with emulsion was *y*_2_ = 0.376*x*_2_ + 1.051, with an *r*-value of 0.993 (*F* = 141.094, *p* = 0.007 < 0.05). The comparison of slopes between these two regression equations yielded an *F*-value of 8.61, indicating a significant difference (*p* = 0.001 < 0.05). Conclusion: Our findings suggest that the resistance factor in *Blattella germanica* treated with beta-cypermethrin nanoemulsion differs from that treated with beta-cypermethrin emulsion. Specifically, the resistance factor of beta-cypermethrin nanoemulsion increased at a slower rate compared to beta-cypermethrin emulsion.

## 1. Introduction

Beta-cypermethrin is a stable pyrethroid compound known for its rapid knockdown effect, high lethality, prolonged persistence, and low toxicity to mammals. It was first synthesized in China in 1988 and has since found widespread use in the control of sanitary pests [1]. Notably, in 2006, the World Health Organization (WHO) recognized it as a vital insecticide for vector control [2]. The mode of action involves altering axon membrane permeability, thereby disrupting axon conduction and leading to the demise of the targeted pests. In this context, insect resistance development is linked to specific resistance genes within the insect population. Over time, continuous exposure to insecticides results in the accumulation of individuals harboring multiple resistance genes, consequently altering the physiological and biochemical mechanisms of these pests [3,4,5,6].

Beta-cypermethrin nanoemulsion represents an insecticide formulation wherein beta-cypermethrin serves as the active ingredient and is prepared using nanoemulsion technology. Nanoemulsion is a technique that harnesses emulsion technology to produce extremely small, suspended particles containing one or more active ingredients, allowing the incorporation of these ingredients into an emulsion form for application.

The beta-cypermethrin nanoemulsion offers several notable advantages: 1. High Efficiency: The fine particles within the nanoemulsion exhibit superior adherence to pests, enhancing the insecticidal effectiveness. 2. Stability: Nanoemulsion is characterized by a uniform suspension with excellent stability, resisting the tendencies of layering or precipitation. 3. Enhanced Permeability: The reduced size of the nanoparticles facilitates improved penetration of the insect’s skin by beta-cypermethrin, ultimately enhancing its efficacy. 4. Environmentally Friendly: During application, nanoemulsion results in reduced environmental pollution and diminished pesticide residues. 

The development of 2.85% beta-cypermethrin nanoemulsion was carried out at the Key Laboratory of Environmental Medicine Engineering, Ministry of Education, School of Public Health, Southeast University (Dingjiaqiao Campus, Gulou District, Nanjing City, Jiangsu Province, China). This research is part of a series of comprehensive studies. In previous studies, we demonstrated that the insecticidal efficacy of nanoemulsion was superior to conventional emulsifiable concentrate. It has been established that beta-cypermethrin nanoemulsion achieves superior insecticidal effects even at lower concentrations than beta-cypermethrin emulsion. However, it remains to be determined whether the speed of resistance generation matches that observed with conventional emulsifiable concentrate, worthy of further investigation. In this manuscript, we further assessed the potential application of beta-cypermethrin nanoemulsion with regard to the speed of resistance development. Additionally, we present suggestions for the reference value of biochemical detection in the context of beta-cypermethrin emulsifiable concentrate. These findings provide the foothold for advancing our understanding of resistance development in this context and guiding future research endeavors. 

## 2. Materials and Methods

### 2.1. Reagent

TritonX–100: Products of Shanghai City Chemical Reagent Factory; Shanghai, China.

p-Nitroanisole (P-NA), p-nitrophenol: Beijing Yucai Fine Chemical Factory products; Beijing, China.

Acetylthiocholine iodide and 5, 5′-dithiobis-nitrobenzoic acid (DTNB): products of Sigma Company; Shanghai, China.

Reduced glutathione (GSH): Products of Huamei Bioengineering Company; Shanghai, China.

Sodium dodecyl sulfate (SDS), ethylenediaminetetraacetic acid (EDTA), dithiothreitol (DTT), 2,4-dinitrochlorobenzene (CDNB): products of Shanghai City Reagent Factory; Shanghai, China.

Reduced coenzyme II (NADPH): product of Roche Company; Phenylmethylsulfonyl fluoride (PMSF): products of Nanjing Shengxing Technology Co., Ltd.; Nanjing, China.

Disodium p-nitrophenyl phosphate, Coomassie brilliant blue: Fluka products;

95% beta-cypermethrin raw powder: Nanjing Pesticide Factory, Jiangsu Province Pesticide Research Institute; Nanjing, China.

2.85% beta-cypermethrin nanoemulsion: The stock solution was developed in Dingjiaqiao Campus, Gulou District, Nanjing City, Jiangsu Province, China. The emulsion exhibits a light yellow, transparent, and uniformly consistent appearance with excellent fluidity. Samples are carefully bottled and sealed for long-term storage at room temperature. In its natural state, the emulsion maintains its transparency without any signs of precipitation or stratification, and its fluidity and emulsifying performance remain unaltered. Notably, the average particle size of this emulsion measures 11.2 nanometers. Prior to use, a diluent is prepared as needed. The remaining reagents were made in China with analytical or chemical purity.

### 2.2. Breeding of Sensitive and Resistant Lines

*Blattella germanica*, which had not been exposed to insecticides for an extended period, was sourced from the Insect Feeding Room of Jiangsu Province Center for Disease Control and Prevention. The susceptible strain for reproduction was selected from those *Blattella germanica* specimens that were knocked down after exposure to insecticides. The exposure of *Blattella germanica* to insecticides was carried out at the Dingjiaqiao Campus, Gulou District, Nanjing City, Jiangsu Province, China. The resistant line was developed using a population screening method. Both beta-cypermethrin emulsion and beta-cypermethrin nanoemulsion, at appropriate concentrations, were used to treat *Blattella germanica* from the same age group, maintaining a mortality rate of approximately 60%. The surviving individuals from this treatment were then used as seed insects for subsequent generation screening, gradually increasing drug resistance levels [7].

### 2.3. Determination of LD50 and Calculation of Its Resistance Factor

The study employed the micro-drop method as specified by WHO [8,9,10]. Beta-cypermethrin nanoemulsion was diluted with an equal proportion of water (acetone CP grade for emulsion) to create six different concentrations, with an additional control group established. A total of 70 healthy adult insects (10 in each group) were anesthetized with ether and positioned on a glass plate. Using the micro-drop method, 1 μL of varying concentrations of the liquid was absorbed by a micro-sampler and applied sequentially to the mid-abdomen, chest, and back plate of the test insects, starting from the lowest to the highest concentrations. Following the application, the test insects were placed in clean jam bottles provided with normal feeding, and the number of deaths among the test insects in each group was recorded after 72 h. *Blattella germanica* that were not exposed to any drugs served as the control group. 

Calculation of resistance factor (R):

$$\begin{array}{l} Resistance\;factor=\frac{assay\;strain\;LD50}{sensitive\;strain\;LD50} \end{array}$$

### 2.4. Determination of Enzyme Activity

Enzyme activity measurement was conducted in a region where the reaction quantity and time displayed a linear relationship. The rate of product formation served as an indicator of enzyme activity, representing the increase in the corresponding product per milligram of protein per unit time [11,12].

#### 2.4.1. Preparation of Enzyme Solution

(1) Preparation of AchE enzyme solution: *Blattella germanica* specimens, having fasted for 24 h post-application, were washed with flowing distilled water for 2 min, and then gently dried on filter paper. The head was isolated, immersed in phosphate buffer (pH = 8.0, 1/15 mol/L) containing 0.5% TritonX-100, homogenized in an ice bath, and centrifuged at 4000× *g* r/min for 15 min. The resulting supernatant was collected as the enzyme solution.
(2) Preparation of GST enzyme solution: Following a 24-h fasting period post-application, *Blattella germanica* specimens were washed with flowing distilled water for 2 min and gently dried on filter paper. At 4 °C, the specimens were dissected, the food within the digestive tract was removed, and an enzyme solution was prepared from the midgut. This solution was created by homogenizing in phosphate buffer (pH 6.5, 0.1 mol/L) in an ice bath and subsequent centrifugation at 10,000× *g* r/min for 15 min. The supernatant was then collected as the enzyme solution.
(3) Preparation of P450-O demethylase solution: Similar to the previous steps, *Blattella germanica* specimens that fasted for 24 h post-application were washed with flowing distilled water for 2 min and gently dried on filter paper. They were dissected at 4 °C, with food removed from the digestive tract. The enzyme solution was prepared from the midgut by adding phosphate-buffered solution (pH 7.8, 0.1 mol/L, containing 1 mmol/L EDTA, 1 mmol/L DTT, and 1 mmol/L PMSF) in an ice bath, followed by centrifugation at 10,000× *g* r/min for 15 min. The supernatant was collected as the enzyme solution.

#### 2.4.2. Determination of Enzyme Activity

(1) AchE activity was determined according to Groun’s modified Ellman method [13]. The final reaction system volume was 0.2 mL. During this process, 100 μL of 1/15 mol/L phosphate buffer (pH 8.0), 50 μL of 0.75 mmol/L substrate (thioacetylcholine iodide), and 50 μL of enzyme solution (adjusted to a protein content of 40~80 μg/mL) were mixed and allowed to react at 30 °C for 15 min. Subsequently, 1.8 mL of DTNB reagent was added, and colorimetric determination was carried out at a 412 nm wavelength.
(2) GST activity was determined as previously described [14]. This involved adding 100 μL of enzyme solution to the reaction system, composed of 2.5 mL of phosphate buffer (pH 6.5, 0.1 mol/L), 0.1 mL of reduced GSH (100 mmol/L), and 20 μL of 2,4-dinitrobenzene (CDNB) acetone solution (50 mmol/L). The mixture was thoroughly combined and left at 25 °C for 20 min. After adding 0.5 mL of SDS (2.5%) and thorough mixing, the absorbance value at 340 nm was recorded using an ultraviolet spectrophotometer every 1 min for a total of 3 min. The enzyme activity [m OD/(mg·min)] was expressed based on the reaction rate, calculated from the absorbance change within 3 min.
(3) Determination of P450-O demethylase activity [15]: For P450-O demethylase activity determination [15], 100 μL of 2.0 mmol/L p-nitroanisole (P-NA), 10 μL of 9.6 mmol/L reduced coenzyme II (NADPH), and 90 μL of enzyme solution were added to each well of a 96-well microtiter plate. The optical density value at 412 nm wavelength was recorded every 25 s for a total of 10 min using a microplate reader. The enzymatic reaction stage was maintained at 30 °C. The reaction rate was calculated based on the optical density change within the range of 0~0.2, and the enzyme activity [nOD/(mg·min)] was expressed as the reaction rate.

#### 2.4.3. Determination of Protein Concentration

The protein concentration was determined following the Bradford method [16]. This involved taking 0.1 mL of the enzyme solution, adding 5 mL of Coomassie brilliant blue, measuring the OD value at 595 nm, and determining the corresponding protein concentration using the standard curve.

### 2.5. Data Processing

Data analysis, including probability regression analysis, correlation regression analysis, variance analysis, and Dunnett’s *t*-test, was conducted using SPSS 22.0 software. Regression curves were generated to visualize the results.

## 3. Results and Analysis

### 3.1. Comparison of Resistance Factor of Different Resistant Strains of Two Insecticides

The LD_50_ values of various resistant strains of *Blattella germanica*, subjected to treatment with beta-cypermethrin nanoemulsion and beta-cypermethrin emulsion, were determined. Subsequently, the resistance factors (R) for these different resistant strains of *Blattella germanica* towards beta-cypermethrin nanoemulsion and emulsion were calculated and are presented in Table 1. 

As observed in Table 1, the resistance factor of *Blattella germanica* steadily increased after treatment with beta-cypermethrin nanoemulsion and emulsion. However, the rate of increase in resistance was lower for beta-cypermethrin nanoemulsion than the emulsion (Table 1). The regression equations describing the relationship between generation and resistance factor for *Blattella germanica* treated with beta-cypermethrin nanoemulsion and emulsion are as follows:

Nanoemulsion: *y*_1_ = 0.091*x*_1_ + 0.991 *r* = 0.990 (*F* = 95.184, *p* = 0.01 < 0.05)

Emulsion: *y*_2_ = 0.376*x*_2_ + 1.051 *r* = 0.993 (*F* = 141.094, *p* = 0.007 < 0.05)

Comparing the slopes of the two regression equations, an *F*-value of 8.61 (*p* = 0.001 < 0.05) was obtained, indicating a statistically significant difference. This suggests that the resistance factor for each generation of *Blattella germanica*, treated with beta-cypermethrin nanoemulsion and emulsion, varies. In conjunction with the data presented in Table 1, it is evident that the resistance factor for beta-cypermethrin nanoemulsion increased at a slower rate compared to that of beta-cypermethrin emulsion.

### 3.2. Comparison of AchE Activity in Blattella germanica Strains with Varied Resistance to Two Insecticides

Following multiple generations, the AchE activity in each generation of *Blattella germanica* treated with beta-cypermethrin nanoemulsion and emulsion is presented in Table 2. The ANOVA results were as follows: for nanoemulsion: *F* = 31.458 (*p* < 0.01); for emulsion: *F* = 117.924 (*p* < 0.01).

Subsequently, Dunnett’s *t*-test was performed for the nanoemulsion group. The results revealed significant differences in the AchE activity of the first and third generations compared to the sensitive strain. This suggests that the AchE activity of the first and third generations of *Blattella germanica* within the nanoemulsion group differed from that of the sensitive strain. Dunnett’s *t*-test was also applied to the emulsion group, showing that all three generations within the emulsion group exhibited statistically significant differences. This implies that enzyme activity increased progressively with each generation (Table 3).

The resistance factor and AchE activity of beta-cypermethrin emulsion-resistant strains of *Blattella germanica* were subjected to fitting and regression analysis. The results of various fitting models are presented in Table 4. Upon analysis, it was observed that only the *p*-value associated with the simple linear function was less than 0.05, indicating that the simple linear function fitting was the most appropriate. Consequently, a preliminary equation for the relationship between AchE activity and the resistance factor of *Blattella germanica* was established as follows: *y* = 15.751151*x* + 63.811891.

### 3.3. Comparison of the Effect of GST Enzyme of Blattella germanica Strains with Different Resistance to Two Insecticides

After several passages, the Glutathione S-transferase (GST) activity of each generation of *Blattella germanica* exposed to beta-cypermethrin nanoemulsion and emulsion is presented in Table 5. The ANOVA results indicate significant differences: for nanoemulsion, *F* = 197.550 (*p* < 0.001), and for emulsion, *F* = 177.079 (*p* < 0.001). Subsequently, Dunnett’s *t*-test was conducted for the nanoemulsion group, revealing that the GST activity of the first and third generations differed significantly from that of the sensitive strain. This suggests that the GST activity in the first and third generations of *Blattella germanica* in the nanoemulsion group deviated from that of the sensitive strain. Similarly, Dunnett’s *t*-test was performed for the emulsion group, showing that all three generations within the emulsion group exhibited statistically significant differences. This implies enzyme activity increased with each successive generation (Table 6).

The resistance factor and GST activity of beta-cypermethrin emulsion-resistant strains in *Blattella germanica* were subjected to fitting and regression analysis, and the results are displayed in Table 7. Upon analysis, it was observed that the *p*-values associated with all three fitting equations were less than 0.05. While the quadratic function yielded the highest *R*^2^ value, it was noted that the introduction of an additional term in the equation did not significantly increase the *R*^2^ value compared to the first-term function. In light of practicality and convenience, it was decided to select the logarithmic function. Consequently, the equation representing the relationship between GST activity and the resistance factor in *Blattella germanica* was established as follows: *y* = 0.226949ln(*x*) + 0.737452. 

### 3.4. Comparison of the Effects of P450-O Demethylase in Blattella germanica Strains with Varied Resistance to Both Insecticides

Following several passages, the P450-O demethylase activity of each generation of *Blattella germanica* exposed to beta-cypermethrin nanoemulsion and emulsion is presented in Table 8. The ANOVA results indicate significant differences: for nanoemulsion, *F* = 49.185 (*p* < 0.01), and for emulsion, *F* = 207.667 (*p* < 0.001). Subsequently, Dunnett’s *t*-test was conducted for the nanoemulsion group. The results revealed that the P450-O demethylase activity of the third generation in both the nanoemulsion and emulsion groups differed significantly from that of the sensitive strain. This suggests that the P450-O demethylase activity increased with each successive generation in both the nanoemulsion and emulsion groups (Table 9).

The resistance factor and P450-O demethylase activity of beta-cypermethrin emulsion-resistant strains in *Blattella germanica* were subjected to fitting and regression analysis. The results of various fitting models are presented in Table 10. Upon analysis, it was observed that the *p*-value was less than 0.05. However, a noteworthy finding was that the *R*^2^ value of the quadratic function equaled 1, which significantly differed from the *R*^2^ values of the first two fitting curves. As a result, the decision was made to preliminarily establish the equation describing the relationship between P450-O demethylase activity and the resistance factor in *Blattella germanica* as follows: *y* = 2.097981 − 0.386465*x* + 0.618365*x*_2_.

### 3.5. Reference Value of Enzyme Activity in Blattella germanica Resistant to Emulsion by Biochemical Method

In accordance with the resistance of *Blattella germanica* to beta-cypermethrin emulsion, an equation relating biological marker and resistance factor was established. The reference values of AchE, GST, and P450-O demethylase in the beta-cypermethrin emulsion group when the resistance factor reached 2.0 are provided in Table 11.

## 4. Discussion

The introduction of the light-stable pyrethroid insecticide permethrin in the late 1970s marked a significant advancement in pest control. It offered several advantages, including high efficiency, a broad spectrum of activity, a good safety profile for humans, prolonged effectiveness, and the ability to excite and repel pests like *Blattella germanica*. Consequently, it quickly gained widespread use in cockroach prevention and control [17,18]. Subsequently, other pyrethroids, such as cypermethrin, deltamethrin, fenvalerate, cyphenthrin, and their formulations emerged. This led to pyrethroid spraying becoming a primary method for *Blattella germanica* control in the 1980s and 1990s. However, the extensive use of synthetic insecticides causes environmental pollution and poses health risks to humans and other animals. Additionally, due to the short life cycle, frequent applications, and strong resistance of *Blattella germanica*, the problem of drug resistance in this pest became increasingly severe [19,20,21,22,23,24,25,26]. 

Detecting insect resistance is a crucial step in preventing its occurrence and spread. For a long time, biological testing methods were employed for resistance testing. However, recent years have seen advancements in understanding resistance mechanisms, leading to the development of biochemical, immunological, and molecular biological testing methods. Among these, the biochemical test method, recommended by WHO as a supplement to biological tests, has gained popularity in field resistance detection [27]. Compared to biological testing, biochemical testing offers quicker and more precise results, allows for the use of stored insect samples, and enables the detection of resistance frequencies in individual insects. Moreover, biochemical testing aids in formulating effective control strategies, improving the speed and accuracy of resistance detection, and promptly predicting resistance risks [28].

In this study, we observed that the rate of resistance development in *Blattella germanica* to beta-cypermethrin nanoemulsion was slower than that of emulsion as passage generations increased. This phenomenon could be attributed to the finer particle size of the nanoemulsion, which enhances its ability to penetrate the epidermal structures of target organisms. Consequently, the nanoemulsion achieved better pesticide efficacy. Furthermore, nanoemulsion facilitated the penetration of active ingredients into animal and plant tissues and was highly efficient in delivering active compounds. Additionally, beta-cypermethrin nanoemulsion enhanced the sensitivity of resistance-related enzymes in *Blattella germanica*, as indicated by previous studies.

Notably, beta-cypermethrin nanoemulsion demonstrated insecticidal efficacy comparable to that of beta-cypermethrin emulsion while using a lower concentration. This insecticidal effect was statistically significant. In a comparative study on the impact of application periods of beta-cypermethrin nanoemulsion and conventional emulsifiable concentrate on enzyme activity related to *Blattella germanica* resistance, we observed a different trend. The recovery rate of enzyme activity in the emulsion was higher than that in the nanoemulsion within 18 days after application in the laboratory. This suggests that the inhibitory effect of the emulsion during the observation period (within 18 days) was not as pronounced as that of the nanoemulsion, potentially explaining the slower development of resistance in beta-cypermethrin nanoemulsion [29].

This study primarily associated beta-cypermethrin resistance in *Blattella germanica* with three enzymes: AChE, GST, and cytochrome P450. Several reasons may contribute to resistance related to these enzymes [30,31,32,33,34]. 1. Gene Mutation: *Blattella germanica* may undergo gene mutations that alter the structure or function of acetylcholinesterase, reducing their sensitivity to beta-cypermethrin. 2. Increased Metabolism/Degradation: *Blattella germanica* can metabolize beta-cypermethrin more rapidly by enhancing metabolic capacity, including increasing the quantity and activity of metabolic enzymes like glutathione transferase and cytochrome P450. This reduces the toxic effects of beta-cypermethrin. 3. Expression Regulation: *Blattella germanica* can regulate gene expression to increase or decrease the expression of cytochrome P450 enzymes. Certain regulatory factors may promote P450 enzyme expression, enhancing *Blattella germanica*’s tolerance to beta-cypermethrin and other pesticides. 

The experimental data also demonstrated that the amount of AchE in the resistant strain was notably higher than in the sensitive strain. This suggests that AchE gene amplification occurs in the resistant population under insecticide selection pressure. During AchE gene amplification, mutations can lead to allosteric AchE, which is less sensitive to pyrethroid drugs like beta-cypermethrin. Changes in AchE quantity and quality can reduce the degree of AchE inhibition. An increase in AchE activity levels in *Blattella germanica* leads to reduced pyrethroid insecticide inhibition and increased resistance [35,36]. Thus, detecting AchE activity and AchE gene structure in *Blattella germanica* serve as effective methods to monitor resistance.

Moreover, our experiments indicate that beta-cypermethrin significantly induces GST in *Blattella germanica* [37]. Comparison between resistant and sensitive strains revealed that GST activity was significantly higher in the resistant strain. This suggests that under insecticide selection pressure, the *GST* gene in the resistant strain is overexpressed, leading to higher GST levels, thereby enhancing the insect’s antioxidant defense. This improved defense mechanism enhances the insect’s ability to recover from pesticide poisoning, making GST activity a valuable indicator in monitoring beta-cypermethrin resistance.

## 5. Conclusions

Previous studies have established that AchE, GST, and P450-O demethylase can serve as biological markers for beta-cypermethrin resistance in *Blattella germanica*. By establishing resistance factor equations for beta-cypermethrin nanoemulsion and emulsion, we can preliminarily assess insect resistance based on the activities of these three enzymes. Resistance factors below 2 indicate sensitivity or weak resistance, whereas resistance factors above 2 indicate the onset of resistance, with resistance factors above 5 indicating moderate resistance [38,39]. In this study, we established equations relating the activity of AchE, GST, and P450-O demethylase to the resistance factor of beta-cypermethrin emulsion in *Blattella germanica* as follows: *y* = 15.751151*x* + 63.811891, *y* = 0.226949ln(*x*) + 0.737452, *y* = 2.097981 − 0.386465*x* + 0.618365*x*_2_. When a resistance factor of 2.0 was used as the threshold for resistance emergence, resistance in the beta-cypermethrin-resistant strain of *Blattella germanica* was significant when the activities of AchE, GST, and P450-O demethylase exceeded 95.31 nmol/(mg·min), 0.90 mOD/(mg·min), and 3.80 nOD/(mg·min), respectively. However, due to the slow development of resistance (a resistance factor of only 1.2784 at the third generation), this study did not observe resistance to beta-cypermethrin nanoemulsion. Therefore, further research is needed to establish the relationship between resistance-related enzyme activity and the resistance factor of *Blattella germanica* to beta-cypermethrin nanoemulsion.

## Figures and Tables

**Table 1:** Resistance factors of different resistant strains of *Blattella germanica* to beta-cypermethrin nanoemulsion and emulsion.

Resistant Strain	Beta-Cypermethrin Nanoemulsion		Beta-Cypermethrin Emulsion	
	LD_50_ (µg/Insect)	Resistance Factor (*R*)	LD_50_ (µg/Insect)	Resistance Factor (*R*)
Sensitive	0.0097	1.0000	0.0338	1.000
First Generation	0.0105	1.0825	0.0500	1.4793
Second Generation	0.0112	1.1546	0.0625	1.8491
Three Generations	0.0124	1.2784	0.0719	2.1272

**Table 2:** AchE activity in different resistant strains of *Blattella germanica* exposed to beta-cypermethrin nanoemulsion and emulsion.

Beta-Cypermethrin Nanoemulsion				Beta-Cypermethrin Emulsion			
Resistant Strain	Resistance Factor (*R*)	Insect Number	AchE Activity nmol/(mg·min)	Resistant Strain	Resistance Factor (*R*)	Insect Number	AchE Activity nmol/(mg·min)
Sensitive (0)	1.0000	10	80.94 ± 1.68	Sensitive (0)	1.0000	10	80.94 ± 1.68
First Generation (1)	1.0825	10	75.36 ± 4.01	First Generation (1)	1.4793	10	85.48 ± 2.26
Second Generation (2)	1.1546	10	83.02 ± 3.32	Second Generation (2)	1.8491	10	91.21 ± 2.87
Third Generations (3)	1.2784	10	87.71 ± 1.85	Third Generation (3)	2.1272	10	99.37 ± 2.29

**Table 3:** Comparison of AchE activity differences between resistant strain and susceptible strain (Dunnett’s *t*-test).

Beta-Cypermethrin Nanoemulsion				Beta-Cypermethrin Emulsion		
Comparison between Groups	Lower 95% Confidence Interval	Mean Difference	Upper 95% Confidence Interval	Lower 95% Confidence Interval	Mean Difference	Upper 95% Confidence Interval
(1)–(0)	−8.7395	−5.5750 *	−2.4105	1.9998	4.5380 *	7.0762
(2)–(0)	−1.0835	2.0810	5.2455	7.7378	10.2760 *	12.8142
(3)–(0)	3.6075	6.7720 *	9.9365	15.8898	18.4280 *	20.9662

“*” indicates a significant difference compared with the control group, *p* < 0.05.

**Table 4:** Fitting of different models between AchE activity and beta-cypermethrin resistance factor.

Model Name	Regression Equation	*F* Value	*p* Value	*R*^2^ Value
simple linear	*y* = 15.751151*x* + 63.811891	30.50020	0.0313 *	0.93846
logarithmic function	*y* = 22.601107ln(*x*) + 79.286586	14.67241	0.0619	0.88004
quadratic function	*y* = 90.365633 − 21.184139*x* + 11.873487*x*_2_	144.63696	0.0587	0.99655

“*” indicates a significant difference compared with the control group, *p* < 0.05.

**Table 5:** GST activity in different resistant strains of *Blattella germanica* exposed to beta-cypermethrin nanoemulsion and emulsion.

Beta-Cypermethrin Nanoemulsion				Beta-Cypermethrin Emulsion			
Resistant Strain	Resistance Factor (*R*)	Insect Number	GST Activity mOD/(mg·min)	Resistant Strain	Resistance Factor (*R*)	Insect Number	GST Activity mOD/(mg·min)
Sensitive (0)	1.0000	10	0.74 ± 0.01	Sensitive (0)	1.0000	10	0.74 ± 0.01
First Generation (1)	1.0825	10	0.58 ± 0.02	First Generation (1)	1.4793	10	0.82 ± 0.02
Second Generation (2)	1.1546	10	0.76 ± 0.03	Second Generation (2)	1.8491	10	0.88 ± 0.01
Third Generations (3)	1.2784	10	0.82 ± 0.02	Third Generations (3)	2.1272	10	0.91 ± 0.02

**Table 6:** Comparison of GST activity differences between resistant and susceptible strains (Dunnett’s *t*-test).

Beta-Cypermethrin Nanoemulsion				Beta-Cypermethrin Emulsion		
Comparison between Groups	Lower 95% Confidence Interval	Mean Difference	Upper 95% Confidence Interval	Lower 95% Confidence Interval	Mean Difference	Upper 95% Confidence Interval
(1)–(0)	−0.1867	−0.1610 *	−0.1353	0.0635	0.0830 *	0.1025
(2)–(0)	−0.0067	0.0190	0.0447	0.1165	0.1360 *	0.1555
(3)–(0)	0.0583	0.0840 *	0.1097	0.1525	0.1720 *	0.1915

“*” indicates a significant difference compared with the control group, *p* < 0.05.

**Table 7:** Fitting of different models of GST activity and beta-cypermethrin resistance factor.

Model Name	Regression Equation	*F* Value	*p* Value	*R*^2^ Value
simple linear	*y* = 0.152950*x* + 0.590485	322.48702	0.0031 *	0.99384
logarithmic function	*y* = 0.226949ln(*x*) + 0.737452	587.26554	0.0017 *	0.99661
quadratic function	*y* = 0.517671 + 0.254233*x* − 0.032559*x*_2_	397.10658	0.0355 *	0.99874

“*” indicates a significant difference compared with the control group, *p* < 0.05.

**Table 8:** Activity of P450-O demethylase in different resistant strains of *Blattella germanica* exposed to beta-cypermethrin nanoemulsion and emulsion.

Beta-Cypermethrin Nanoemulsion				Beta-Cypermethrin Emulsion			
Resistant Strain	Resistance Factor (*R*)	Insect Number	P450-O Activity nOD/(mg·min)	Resistant Strain	Resistance Factor (*R*)	Insect Number	P450-O Activity nOD/(mg·min)
Sensitive (0)	1.0000	10	2.33 ± 0.07	Sensitive (0)	1.0000	10	2.33 ± 0.07
First Generation (1)	1.0825	10	2.50 ± 0.15	First Generation (1)	1.4793	10	2.88 ± 0.19
Second Generation (2)	1.1546	10	2.86 ± 0.15	Second Generation (2)	1.8491	10	3.50 ± 0.15
Third Generation (3)	1.2784	10	3.06 ± 0.20	Third Generation (3)	2.1272	10	4.08 ± 0.23

**Table 9:** Comparison of P450-O demethylase activity differences between resistant and susceptible strains (Dunnett’s *t*-test).

Beta-Cypermethrin Nanoemulsion				Beta-Cypermethrin Emulsion		
Comparison between Groups	Lower 95% Confidence Interval	Mean Difference	Upper 95% Confidence Interval	Lower 95% Confidence Interval	Mean Difference	Upper 95% Confidence Interval
(1)–(0)	0.0141	0.1780 *	0.3419	0.3706	0.5540 *	0.7374
(2)–(0)	0.3651	0.5290 *	0.6929	0.9926	1.1760 *	1.3594
(3)–(0)	0.5681	0.7320 *	0.8959	1.5766	1.7600 *	1.9434

“*” indicates a significant difference compared with the control group, *p* < 0.05.

**Table 10:** Fitting of different models between P450-O demethylase activity and beta-cypermethrin resistance factor.

Model Name	Regression Equation	*F* Value	*p* Value	*R*^2^ Value
simple linear	*y* = 1.537104*x* + 0.715077	113.43873	0.0087 *	0.98267
logarithmic function	*y* = 2.232321ln(*x*) + 2.213410	33.70863	0.0284 *	0.94399
quadratic function	*y* = 2.097981 − 0.386465*x* + 0.618365*x*_2_	1,217,237.88239	0.0006 *	1.00000

“*” indicates a significant difference compared with the control group, *p* < 0.05.

**Table 11:** Reference values of related enzyme activities of *Blattella germanica* when detecting its resistance to beta-cypermethrin emulsion by biochemical method.

Resistance Factor (*R*)	AchE Activity nmol/(mg·min)	GST Activity mOD/(mg·min)	P450-O Demethylase nOD/(mg·min)
2.0	95.31	0.89	3.80

## Data Availability

No new data created, but if you need our data you can contact the first author.

## References

[1] Jiang Z.K., Zhao X.Z., Yin X.Y. (1992). Efficacy of beta-cypermethrin against health pests. Med. Anim. Control.

[2] Wang Y. (2021). WHO recommended formulation of sanitary pesticides for controlling important vectors and its application. Pestic. Sci. Manag..

[3] Zeng X., Yu C., Gao X. (2004). Biochemical characteristics of cytochrome P450 in resistant and susceptible strains of blattella germanica. China J. Vector Biol. Control.

[4] Agosin M., Kerkut G.A., Gilbert L.I. (1985). Role of microsomal oxidations in insecticide degradation. Comprehensive Insect Physiology, Biochemistry and Pharmacology.

[5] Bureau of Disease Control and Prevention, Ministry of Health (2007). Manual of Malaria Control.

[6] Zeng L., Sun D., Zhao W. (2020). Resistance of Culex pipiens quinquefasciatus and Anopheles dirus to pyrethroid insecticides. China J. Vector Biol..

[7] Wu Y., Shen J., You Z. (1994). Breeding of resistant and susceptible strains of Helicoverpa armigera to fenvalerate. Acta Entomol. Sin..

[8] Ma L., Dou F., Cao R. (2021). Investigation on resistance of Musca domestica and its control strategy in Chengdu City. Med. Anim. Control.

[9] Xu Z., Cao L. (2007). Investigation on resistance of housefly in Zhangjiagang City. J. Med. Anim. Control.

[10] Li F., Chen M. (2021). Efficacy of 9 insecticides against blattella germanica on passenger train. China J. Vector Biol. Control.

[11] Michael E.S., Walid K. (1997). Changes in an insecticide-resistant field population of blattella germanica (Dictyoptera: Blattelidae) after expose to an insecticide mixture. Econ. Entomol..

[12] Valles S.M., Koehler P.G. (1999). Comparative insecticide susceptibility and detoxification enzyme activities among pestiferous Blattodea. Comp. Biochem. Physiol. Part C.

[13] Gorun V., Proinov L., Baltescu V. (1978). Modified Ellman procedure for assay of cholinesterases in crude enzymatic preparations. Anal. Chem..

[14] Wu X.F., Deng J.H. (2019). Effect of continuous use of pesticides on the development of resistance of *Myzus persicae*. China J. Tob..

[15] Xia S., Wu Z. (2000). Molecular Toxicology Basis.

[16] Bradford M.M. (1976). A rapid and sensitive method for the quantitation of microgram quantites of protein utilizing the principle of protein dybinging. Anal. Chem..

[17] Ren S., Liu Y., Yang J. (2020). Toxic effect of organophosphorus pesticide dimethoate on fish. J. Inn. Mong. Univ. Natl. (Nat. Sci. Ed.).

[18] Zhai D.S., Zhao Y., Du B.S. (2020). Acute toxicity test of paraquat in mice. West China J. Pharm. Sci..

[19] Zhao Z., Huo X. (2018). Insecticide resistance mechanism and control strategy of blattella germanica. Chin. J. Health Insectic..

[20] Tang Y., Sun B.X., Zhang L.M. (2018). Resistance test of blattella germanica to conventional insecticides in Changchun City. China Health Insectic..

[21] Yu X. (2018). Research progress of blattella germanica surveillance. China J. Vector Biol. Control.

[22] Ge C., Wang L., Zhang W. (2017). Investigation on resistance of blattella germanica to five insecticides in Fushun City. China Health Insectic..

[23] Yu X., Zeng J., Liu J. (2021). Investigation on population density and infestation status of cockroaches in Neijiang City from 2017 to 2019. Chin. J. Health Insectic..

[24] Li L., Hu Y., Yu J. (2022). Surveillance of cockroach density and investigation of insecticide resistance of *Blattella germanica* in Sichuan Province. Zhonghua Health Insectic. Instrum..

[25] Liu Y., Liu H., Leng P. (2020). Laboratory efficacy of gel baits with four different active ingredients against *Blattella germanica*. Chin. J. Vector Biol. Control.

[26] Kobayashi M., Komatsu N., Ooi H.K. (2021). Prevalence of *Blatticola blattae* (Thelastomatidae) in blattella germanicaes *Blattella germanica* in Japan. J. Vet. Med. Sci..

[27] Su S., Ye B. (1999). Modern Medical Entomology.

[28] Ma Y., Huo X., Ma M. (2014). Inhibitory effect of beta-cypermethrin on acetylcholinesterase of blattella germanica. China J. Vector Biol. Control.

[29] Zhang G. (2022). Insecticide resistance and its management. Anhui Agric. Sci..

[30] Hou W., Xin J., Lu H. (2021). Resistance development characteristics of reared blattella germanica (Blattodea:Blattellidae) to chlorpyrifos. Sci. Rep..

[31] Li T., Wu Y., Liu Q. (2022). Analysis of monitoring results of insecticide resistance of *Blattella germanica* in Zhejiang Province in 2018. Chin. J. Vector Biol. Control.

[32] Zhang C., Zhang J. (2022). Investigation on resistance of *Blattella germanica* to commonly used insecticides in Pingdingshan City from 2018 to 2020. China Health Insectic. Instrum..

[33] Lu C., Zhang J., Zhao J. (2022). Study on the change of resistance of *Blattella germanica* in Yangpu District of Shanghai City from 2014 to 2020. Shanghai J. Prev. Med..

[34] Chao G., Lv W., Lu B. (2021). Cockroach surveillance and resistance analysis of *Blattella germanica* in Xianyang City in 2018. Chin. J. Health Insectic..

[35] Oppenoorth F.J., Kerkut G.A., Gibert I. (1985). Biochemistry and genetics of insecticide resistance. Comprehensive Insect Physiology. Biochemistry and Pharmacology.

[36] Fournier D., Mutero A. (1994). A modification of acetylcholinesterase as a mechanism of resistance to insecticides. Comp. Biochem. Physiol..

[37] Huo X., Yu G., Ma Y. (2021). Induction of glutathione peroxidase by cypermethrin in blattella germanica. China Public Health.

[38] Cochran D.G. (1989). Monitoring for Insecticide Resistance in Filed-Collected Strains of German Cochroach (Dictyoptera:Blattellidate). Econ. Entomol..

[39] Zhou H., Ma Y., Chen Z. (2021). Resistance surveillance of blattella germanica to 5 insecticides in Wuxi city. Jiangsu Prev. Med..

